# Which Oxford Knee Score level represents a satisfactory symptom state after undergoing a total knee replacement?

**DOI:** 10.1080/17453674.2020.1832304

**Published:** 2020-10-13

**Authors:** Lina H Ingelsrud, Berend Terluin, Kirill Gromov, Andrew Price, David Beard, Anders Troelsen

**Affiliations:** a Department of Orthopaedic Surgery, Copenhagen University Hospital Hvidovre, Copenhagen, Denmark;; b Department of General Practice and Elderly Care Medicine, VU University Medical Center, Amsterdam, Netherlands;; c Nuffield Department of Orthopaedics, Rheumatology and Musculoskeletal Sciences, University of Oxford, UK

## Abstract

Background and purpose — Meaningful interpretation of postoperative Oxford Knee Score (OKS) levels is challenging. We established Patient Acceptable Symptoms State (PASS) and Treatment Failure (TF) values for the OKS in patients undergoing primary total knee replacement (TKR) in Denmark.

Patients and methods — Data from patients undergoing primary TKR between February 2015 and January 2019 was extracted from the arthroplasty registry at the Copenhagen University Hospital, Hvidovre in Denmark. Data included 3, 12, and 24 months postoperative responses to the OKS and 2 anchor questions asking whether they considered their symptom state to be satisfactory, and if not, whether they considered the treatment to have failed. PASS and TF threshold values were calculated using the adjusted predictive modeling method. Non-parametric bootstrapping was used to derive 95% confidence intervals (CI).

Results — Complete 3, 12, and 24 months postoperative data was obtained for 187 of 209 (89%), 884 of 915 (97%), and 575 of 586 (98%) patients, with median ages from 68 to 70 years (59 to 64% female). 72%, 77%, and 79% considered as having satisfactory symptoms, while 6%, 11%, and 11% considered the treatment to have failed, at 3, 12, and 24 months postoperatively, respectively. OKS PASS values (CI) were 27 (26–28), 30 (29–31), and 30 (29–31) at 3, 12, and 24 months postoperatively. TF values were 27 (26–28) and 27 (26–29) at 12 and 24 months postoperatively.

Interpretation — The OKS PASS values can be used to guide the interpretation of TKR outcome and support quality assessment in institutional and national registries.

The patient perspective on outcome of total knee replacement (TKR) is captured with patient-reported outcome measures (PROMs) (Price et al. 2018). The Oxford Knee Score (OKS) measures the degree of knee pain and functional status of the knee on a scale ranging from 0 to 48 (worst to best score) (Dawson et al. 1998). Registry-based data suggest that 6-months postoperative OKS results are on average 36 points (NHS 2020). However, judging whether the outcome of surgery was successful or not can be challenging, because it is not clear which symptom level patients consider to be satisfactory. The Patient Acceptable Symptom State (PASS) concept was defined by Tubach et al. (2005) as the score on a PROM above which patients consider themselves well. The contrary concept, Treatment Failure (TF), was introduced for patients undergoing ACL reconstruction, to define patients who consider their symptom levels unsatisfactory to a degree that they find the treatment has failed (Ingelsrud et al. 2015).

Suggested satisfaction thresholds for the OKS range from 30 to 38 points after knee replacement (Judge et al. 2012, Keurentjes et al. 2014, Petersen et al. 2017). The time-points evaluated in these studies were either 6 months or shorter/longer than 3 years postoperatively. A dichotomized visual analogue scale (VAS) or a numeric rating scale (NRS) was used as anchor question to measure patients’ satisfaction. However, having the patients’ explicit judgements of whether they have reached a satisfactory symptom state or not after surgery is necessary to derive credible PASS values. Moreover, interpretation characteristics of PROMs are context dependent (Tubach et al. 2007), which highlights the relevance of evaluateing the time-dependency of PASS values for the OKS after TKR. We therefore defined PASS and TF values for the OKS at 3 months, and 1 and 2 years after a TKR.

## Patients and methods

Data from patients undergoing primary TKR due to primary or secondary OA between February 2015 and January 2019 were extracted from the arthroplasty registry at the Copenhagen University Hospital, Hvidovre in Denmark. Registry data were predominantly collected electronically, with patients responding to an electronic questionnaire during their pre-surgical visit to the hospital. Links to follow-up questionnaires were sent by email at 3, 12, and 24 months postoperatively. Paper versions were sent to patients without an e-mail address and to all patients not responding to an electronic reminder. Patients’ BMI was calculated using self-reported preoperative height and weight while the ASA score and Kellgren and Lawrence classification of radiographic OA was reported to the registry by the operating surgeon.

Questionnaire data for this study included the OKS and 2 additional anchor questions that were responded to postoperatively. The first question asked, “Taking into account all the activities you have during your daily life, your level of pain, and also your functional impairment, do you consider that your current state is satisfactory?” (yes/no). If the patients answered that they did not have a satisfactory symptom state, they were asked the second question: “Would you consider your current state as being so unsatisfactory that you think the treatment has failed?” (yes/no). Administration of these anchor questions in the registry was initiated at different points in time for the 3 follow-up time-points, which is why the numbers of eligible patients differ across the 3 follow-up time-points.

### Statistics

Patient characteristics are reported as median (interquartile range [IQR]) for non-normally distributed continuous variables and number (proportion) for categorical variables. Postoperative OKS distributions across anchor response categories were investigated with boxplots. The association between the postoperative OKS score and anchor responses were investigated with Spearman’s correlation. R version 3.4.1 (https://www.r-project.org/) was used for analyses.

PASS and TF threshold values for the OKS were calculated using the predictive modeling method (Terluin et al. 2015), which was originally developed to estimate minimal important change thresholds, but can also be used to estimate thresholds in cross-sectional data. The method is based on logistic regression, with the PASS and TF anchors as dependent variables and postoperative OKS as the independent variable. The threshold is the OKS score that corresponds to a likelihood ratio of 1. With a likelihood ratio of 1, the posttest odds of having a satisfactory symptom state are the same as the pretest odds of having a satisfactory symptom state. The predictive modeling method identifies thresholds that are close to optimal ROC cut-offs with greater precision than ROC analysis (Terluin et al. 2015). However, both thresholds tend to be biased if the proportions of the dependent variable are unequally distributed, resulting in overestimation of the threshold if the proportion having a satisfactory state is greater than 50% or underestimation if the proportion is smaller than 50%. We therefore applied an adjustment to the threshold for unequal proportions of patients, with the equation proposed by Terluin et al. (2017):

$${}^{PASSadjusted=PASSpred–(0.090+0.103}{*}^{Cor)*SD*log-odds(sat)}$$

In this equation, *Cor* is the point biserial correlation between the postoperative OKS and the anchor, *SD* is the SD of the postoperative OKS, and *log-odds(sat)* is the natural logarithm of (proportion with satisfactory symptom state/[1 – proportion with satisfactory symptom state]). Non-parametric bootstrapping (n = 1,000) was used to derive 95% confidence intervals (CI) and is reported as 0.025–0.975 quantiles.

Subgroup analyses were performed to investigate the effect of preoperative severity level on the PASS and TF values. We calculated stratified PASS and TF values for high-severity (lower OKS score) and low-severity (higher OKS score) subgroups that were split by the median preoperative OKS score. Furthermore, to generate CIs around the differences in PASS and TF values for the high- and low-severity subgroups, we median split 1,000 non-parametric bootstrap samples. Severity subgroup PASS and TF values were considered to be statistically different if the 95% CI of the differences did not include 0. For comparison with previous studies, we calculated cut-offs with the receiver operating characteristics (ROC) statistics. The cut-off was determined according to the Youden principle as the point yielding the largest sum of sensitivity and specificity (Youden 1950). We expected the Youden threshold to be close to the predictive modeling threshold and higher than the adjusted predictive modeling threshold if the proportion satisfactory state was greater than 50%. We also applied an 80% specificity rule, since other studies have suggested that thresholds determined as the point with the highest degree of sensitivity and at least 80% specificity improve comparability across studies (Aletaha et al. 2009).

### Ethics, funding, and potential conflicts of interest

The local arthroplasty registry was approved by the Danish Data Protection Agency (Journal number HVH-2012-048). In Denmark, approval from the ethical committee is not required for register-based studies involving only questionnaire data. The study was fully funded by the Department of Orthopaedic Surgery at the hospital. The authors declare no potential conflicts of interest in relation to this study.

## Results

**Figure 1.**
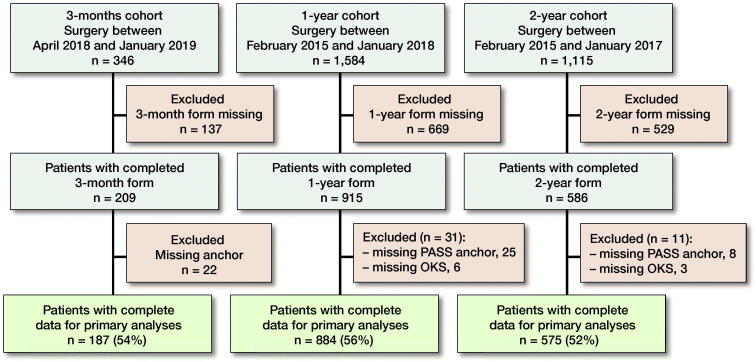
Flow chart of patient enrollment.

Of the eligible patients in the registry, complete data was obtained from 187 (54%), 884 (56%), and 575 (52%) patients at 3 months, 1 year, and 2 years’ follow-up (Figure 1). At surgery, the median age was 68–70 years and 59–64% were female (Table 1).

**Table 1.** Patient demographics and preoperative characteristics. Values are median (2.5–97.5% quantile range) and count (percentage)

	3 months	1 year	2 years
Factor	n = 187	n = 884	n = 575
Age	70 (60–75)	69 (61–74)	68 (61–74)
Female sex	110 (59)	545 (62)	365 (64)
BMI	28 (25–32)	29 (26–33)	29 (26–33)
missing data	–	6	7
ASA			
1	22 (12)	115 (13)	78 (14)
2	132 (71)	623 (70)	398 (69)
3	33 (18)	144 (16)	98 (17)
4	–	2 (0)	1 (0)
Kellgren and Lawrence grade			
1	1 (1)	5 (1)	4 (1)
2	12 (6)	91 (10)	61 (11)
3	70 (37)	377 (43)	251 (44)
4	104 (56)	411 (47)	259 (45)
Oxford Knee Score	24 (18–29)	22 (17–27)	22 (17–27)
missing data	33	251	158
EQ5D index	0.72	0.68	0.66
	(0.63–0.72)	(0.56–0.72)	(0.56–0.72)
missing data	32	254	160

At 3 months postoperatively, 72% considered themselves to have satisfactory symptoms, while 6% considered their symptom state as being so unsatisfactory that they considered the treatment to have failed. The proportions of patients who were satisfied with their symptom level were 77% and 79% at 1 and 2 years postoperatively, while 11% considered the treatment to have failed (Table 2).

**Table 2.** Proportions of patients achieving a satisfactory symptom level, considering treatment failure, or neither at 3 months, 1 year, and 2 years after surgery. Values are count (percentage)

	3 months	1 year	2 years
Factor	n = 187	n = 884	n = 575
Satisfactory symptom level	135 (72)	684 (77)	456 (79)
Neither satisfactory symptoms			
nor treatment failure	39 (21)	99 (11)	52 (9)
Treatment failure	12 (6)	93 (11)	63 (11)
Treatment failure anchor missing	1 (1)	8 (1)	4 (1)

**Figure 2.**
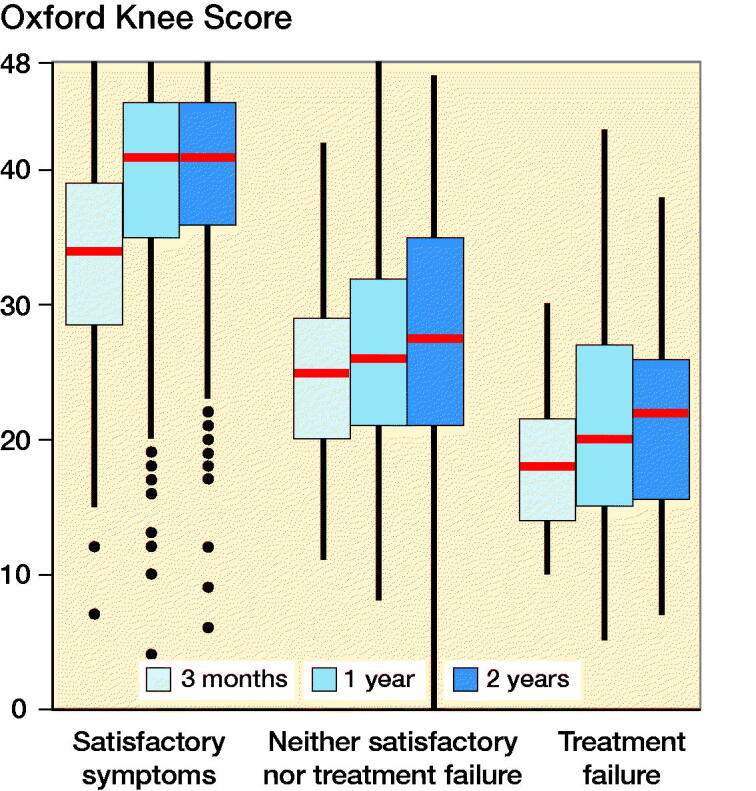
Oxford Knee Score (OKS) distributions at 3, 12, and 24 months postoperatively for patients with satisfactory symptoms, considering the treatment to have failed, or neither. Red bars present the median, the box the interquartile range (IQR), and the whiskers the maximum and minimum scores within 1.5 _*_ IQR from the box. Outliers are values beyond 1.5 _*_ IQR from the box.

Postoperative OKS were in general higher for patients considering their symptom level to be satisfactory, in comparison with those considering the treatment to have failed or neither (Figure 2). Spearman’s correlation between the postoperative OKS and the classification “satisfactory symptoms,” “neither satisfactory nor treatment failure,” or “treatment failure” was 0.52 at 3 months, 0.59 at 12 months, and 0.58 at 24 months.

When adjusting the predictive modeling method PASS values for the high proportion having satisfactory symptoms, OKS PASS values were 27 at 3 months, 30 at 12 months, and 30 at 24 months after TKR. TF values were 27 points at 12 months, and 27 at 24 months postoperatively (Table 3). At 3 months, the absolute number of patients considering the treatment to have failed (n = 12) was too low to calculate TF values.

**Table 3.** Patient Acceptable Symptom State (PASS) and treatment failure (TF) cut-off values calculated with the adjusted predictive modeling method for the Oxford Knee Score at 3, 12, and 24 months after a total knee replacement

Follow-up	n	PASS value (95% CI) **^a^**	n	TF value (95% CI) **^a^**
3 months	187	27 (26–28)		–
12 months	884	30 (29–31)	876	27 (25–28)
24 months	575	30 (29–31)	571	27 (26–29)

**^a^** 95% confidence intervals (CI) are the 0.025–0.975 quantiles of the 1,000 bootstrap threshold values.

Subgroup analyses showed that mean bootstrapped PASS and TF values were 4–6 points higher in the low-severity subgroups in comparison with the high-severity subgroups at 12 and 24 months, respectively (Table 4). Further, the adjusted predictive modeling method yielded smaller PASS and TF values than the ROC method and had smaller CIs for all follow-up time-points (Supplementary data).

**Table 4.** Baseline dependency of Patient Acceptable Symptom State (PASS) and Treatment Failure (TF) cut-off values calculated with the adjusted predictive modeling method for the Oxford Knee Score at 12 and 24 months after total knee replacement

Factor	Mean threshold value **^a^** (95% CI **^b^**) High-severity subgroup **^c^**	Mean threshold value **^a^** (95% CI **^b^**) Low-severity subgroup **^c^**	Mean difference in threshold value (95% CI)
PASS			
12 months	29 (28–30)	33 (32–34)	–4 (–5 to –2)
24 months	27 (26–29)	34 (32–35)	–6 (–9 to –4)
TF			
12 months	24 (22–26)	31 (29–32)	–6 (–9 to –3)
24 months	25 (23–27)	30 (28–32)	–6 (–9 to –3)

**^a^** PASS and TF values are presented as the mean of 1,000 bootstrap threshold values.

**^b^** 95% confidence intervals (CI) are the 0.025–0.975 quantiles of the 1,000 bootstrap threshold values.

**^c^** High- and low-severity subgroups were generated by splitting each bootstrap sample by the median preoperative OKS score.

## Discussion

We estimated OKS PASS values at 3 months, 1, and 2 years after a total knee replacement. A PASS value can be interpreted as the threshold between what the average patient would consider a satisfactory state and what they would consider a non-satisfactory state. Our finding that PASS values increased by approximately 3 points from 3 months to 1 and 2 years postoperatively suggests that patients accept lower levels of knee functional status in the shorter term than they do in the longer term after surgery. Furthermore, we found that PASS values varied with the preoperative functional level.

The PASS values we derived lie in the lower range of proposed cut-offs between 30 and 38 points for the OKS that previously have been suggested to reflect satisfaction with knee replacement (Judge et al. 2012, Keurentjes et al. 2014, Petersen et al. 2017, Hamilton et al. 2018). The variations in values from previous studies may be caused by differences in statistical methods, anchor questions used, and follow-up time-points. Our PASS value of 27 at 3 months postoperatively is lower than the values of 30 and 35 that were previously found at 6 months postoperatively (Judge et al. 2012, Petersen et al. 2017). Furthermore, our PASS values of 30 at 12 and 24 months are lower than any of the other previously published values using the same or longer follow-up time-periods (Keurentjes et al. 2014, Hamilton et al. 2018). Increasing PASS values with increasing follow-up time-points were previously suggested by Keurentjes et al. (2014), who found OKS PASS values of 34 for patients < 3 years and 38 for patients > = 3 years postoperatively. In contrast, we found an increase in PASS values from 3 to 12 months, but almost similar PASS values at 12 and 24 months postoperatively.

Differences in statistical approaches hinder direct comparison of PASS values. We have shown in this study that different methods yield different results. The PASS values we derived with other methods were 2–5 points higher than those derived using the primary analysis method. The overestimation of PASS values calculated with the ROC method is associated with the proportions of patients with satisfactory symptom levels exceeding 50% (Terluin et al. 2020). Advantages of using the adjusted predictive modeling method include that we are able to overcome the issue of biased PASS values in the direction of the largest group (Terluin et al. 2017). Furthermore, smaller CIs reflect greater precision of this method in comparison with the more traditional ROC method.

Another particular strength of our study is the dichotomous anchor question used. All previous studies have anchored the postoperative OKS on dichotomized 0–100 VAS or 0–10 NRS measuring satisfaction with the outcome. These anchors were dichotomized using thresholds of ≥ 5 on the NRS (range 0 to 10) (Keurentjes et al. 2014), and ≥ 50 or ≥ 70 on the VAS scales (range 0 to 100) (Judge et al. 2012, Petersen et al. 2017). A shortcoming of previous studies is therefore that the dichotomization thresholds are seemingly arbitrarily chosen. Judge et al. (2012) tested the effect of varying the cut-off on the satisfaction VAS and concluded that the PASS values varied by only 3 points with the choice of cut-off on the VAS scale. However, considering that the concept PASS reflects the threshold level of symptoms that patients consider satisfactory, the experts to judge are the patients themselves. An advantage of our PASS anchor question is therefore that the classification of being in a satisfactory symptom state or not relies totally on the patients’ own judgement.

The presented TF thresholds were only 3 points lower than the PASS thresholds. The small difference implies that the undecided area between considering treatment success and treatment failure is not prominent and, in turn, suggests that treatment outcome can be dichotomized into successful and not successful outcome using the PASS values. Depending on the question patients are asked and the definition of treatment success, the proportion of patients with successful outcome after knee replacement varies (Hamilton et al. 2018). A possible limitation of the PASS anchor item we used is that it does not specifically ask about the current state of the operated knee. However, we believe that patients actually do reflect on their knee status when responding to this anchor item, since this question is asked subsequently after knee-specific PROMs. A qualitative study approach would be necessary to clarify the anchor items’ degree of content validity and to further investigate the use of PASS and TF anchor questions in reflecting postoperative treatment success.

We found that PASS and TF values were baseline dependent. Patients with lesser symptoms preoperatively required fewer symptoms to consider being in a satisfactory symptom state, in comparison with patients with worse preoperative symptom levels. In other words, patients with higher OKS scores preoperatively required higher OKS scores at the postoperative follow-up time points to consider their symptom levels satisfactory. The differences in 12- and 24-month PASS values were 3 and 6 points, between patients with higher and lower preoperative severity levels. Baseline dependency in satisfaction thresholds was also previously found in Judge and colleagues’ (2012) study of the OKS, and also the Oxford Hip Score in patients undergoing total hip replacement (Arden et al. 2011). That PASS values vary with preoperative severity levels underlines the importance of considering whether preoperative patient characteristics are comparable, when applying PASS values to interpret OKS results from other data sources.

PASS values can be used as responder criteria. In clinical trials, such responder analyses, presenting the proportions of patients with postoperative OKS exceeding the PASS values in each trial arm, may increase the interpretation of treatment success, as was previously exemplified in patients undergoing ACL reconstruction (Roos et al. 2019). Likewise, the interpretation of registry-based PROM collection or cohort study results can be improved in a similar manner, and may contribute to quality assessment of TKR based on institutional or national registries. From a clinical perspective, PASS values serve as group-based reference values and should not be misinterpreted as representing every individual patient. These values that represent thresholds for the average patient may serve as comparability values from a reference population in the postoperative management, rather than fixed goals of treatment for every patient. Furthermore, PASS values and proportions of responders may be helpful to patients and clinicians in their shared-decision dialogue when deciding whether to undergo surgery and to leverage expectations. Importantly, the finding that PASS values are baseline dependent stresses the fact that application in other populations and cohorts must be done with careful consideration of the patient characteristics’ comparability to our study cohort.

The study data, collected in a single public hospital in Denmark, possibly limits the generalizability of the PASS values. Furthermore, we had complete data from only just above 50% of the patients who underwent surgery in the study period, which may introduce selection bias. However, considering the hospitals’ uptake area, covering both rural and urban geographical areas, and patient characteristics from our sample mirroring the characteristics from the nationwide Danish Knee Arthroplasty Register with regard to age and sex distribution, this supports the representativeness of our study population in a Danish context (Odgaard et al. 2019). Whether the suggested PASS values are applicable in other countries and cultures needs to be established using data from large-scale international registries. Furthermore, we included only patients undergoing a TKR, while unicompartmental knee replacement is increasingly used in Denmark (Henkel et al. 2019). PASS values are considered context dependent, and specific to the patient population and intervention under study (Tubach et al. 2007). Whether PASS values differ with regard to surgical strategy for knee OA remains unanswered.

In conclusion, in patients undergoing TKR, the symptom level patients experience as satisfactory is higher in the shorter term than in the longer term postoperatively. Furthermore, across all investigated follow-up time-points, thresholds for considering their symptom levels as satisfactory are higher for patients who have lower symptom levels preoperatively. The established PASS values can be used to guide the interpretation of TKR outcome when measured with the OKS. Future studies should investigate the external validity of the derived PASS values.

## Supplementary Material

Supplemental Material

## Supplementary data

PASS and TF values calculated with Receiver Operating Characteristics analyses are available as supplementary data in the online version of this article, http://dx.doi.org/10.1080/17453674.2020.1832304
