# Supplementary material for: Which Oxford Knee Score level represents a satisfactory symptom state after undergoing a total knee replacement?
Source: Acta Orthop. 2020 Oct 13;92(1):85–90. doi: 10.1080/17453674.2020.1832304 (PMC7919874; doi:10.1080/17453674.2020.1832304)
Supplement: Supplemental Material [file IORT_A_1832304_SM6401.pdf]

Supplementary data

PASS and TF values calculated with Receiver Operating Characteristics analyses

Methods: In addition to calculating PASS and TF values with the adjusted predictive modeling method (Terluin et al. 2017), analyses were performed using the receiver operating characteristics (ROC) statistics to enable comparison with previous studies. We determined ROC cut-offs in two ways. First, according to the Youden principle as the point yielding the largest combination of sensitivity and specificity (Youden 1950). Second, we applied an 80% specificity rule, since other studies have suggested that thresholds determined as the point with the highest degree of sensitivity and at least 80% specificity improves comparability across studies (Aletaha et al. 2009).

Results: We found that for each separate time-point, PASS and TF values varied by 2–5 points with different statistical methods (Tables S1 and S2 and Figure S1). Threshold values were lowest when calculated with the adjusted modeling method.

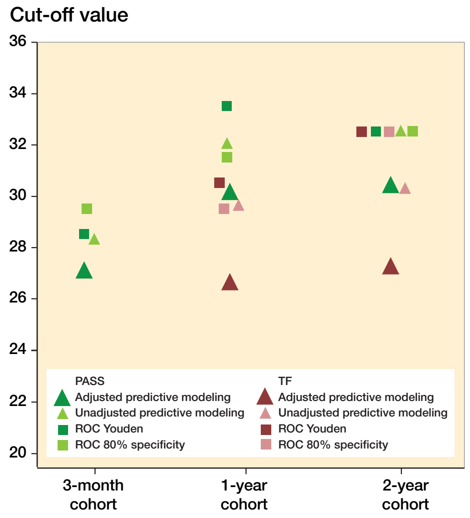

Figure S1. Patient Acceptable Symptom State (PASS) values (green markers) and Treatment Failure (TF) values (red markers) for the 3-, 12-, and 24-month groups calculated with adjusted and unadjusted predictive modeling, and receiver operating characteristic (ROC) statistics using the Youden threshold as cut-off and an 80% specificity rule.

Table S1. Patient Acceptable Symptom State (PASS) values and 95% confidence intervals (CI) for the Oxford Knee Score (OKS) in patients undergoing total knee replacement

Factor	n	Adjusted predictive modeling PASS (CI)	Predictive modeling PASS (CI)	ROC PASS Youden Index (CI)	AUC	Spec	Sens	ROC PASS 80% specificity rule (CI)	Spec	Sens
3 months	187	27 (26–28)	28 (27–30)	29 (25–33)	0.83	0.77	0.75	30 (28–33)	0.81	0.70
12 months	884	30 (29–31)	32 (31–33)	34 (31–37)	0.91	0.88	0.80	32 (31–34)	0.80	0.85
24 months	575	30 (29–31)	33 (32–33)	33 (30–36)	0.91	0.82	0.86	33 (30–36)	0.82	0.86

AUC: area under the curve. Spec: specificity. Sens: sensitivity.
Primary PASS results are the adjusted predictive modeling PASS values. Results are also shown using unadjusted predictive modeling and receiver operating characteristic (ROC) analyses.

Table S2. Treatment Failure (TF) values and 95% confidence intervals (CI) for the Oxford Knee Score (OKS) in patients undergoing total knee replacement

Factor	n	Adjusted predictive modeling TF (CI)	Predictive modeling TF (CI)	ROC TF Youden Index (CI)	AUC	Spec	Sens	ROC TF 80% specificity rule (CI)	Spec	Sens
3 months	186	–	–	–	–	–	–	–	–	–
12 months	876	27 (25–28)	30 (29–31)	31 (27–34)	0.90	0.86	0.80	31 (27–33)	0.86	0.80
24 months	571	27 (26–29)	30 (29–31)	33 (28–33)	0.93	0.95	0.80	28 (26–31)	0.84	0.88

AUC: area under the curve. Spec: specificity. Sens: sensitivity.
Primary TF results are the adjusted predictive modeling TF values. Results are also shown using unadjusted predictive modeling and receiver operating characteristic (ROC) analyses.
